# Mr. Jones, on the Effects of Arsenic

**Published:** 1810-05

**Authors:** Thomas Jones

**Affiliations:** Member of the Royal College of Surgeons, London. Howden


### *To the Editors of the Medical and Physical Journal.*

GENTLEMEN,

ANN WHITE, a stout young woman, walked with her companions, on Wednesday October the 17th, 1809, from Selby fair to Barmly, a distance of seven miles, where she resided, apparently in good health and spirits. She arrived at home about two o'clock in the afternoon, and was observed to be unwell about four, complaining of a violent pain at her stomach, and vomiting frequently; she was afterwards much purged, and all the time desired to have repeated draughts of water, which I believe was warm when taken; she had no medical attendance, and died about two o'clock in the morning. It being afterwards suspected that she had taken, or some person given her, poison, an inquest was held on Saturday the 21st, about noon, when my partner Mr. Wikeley, and myself, were suddenly summoned to open the body. We found it free from putrefaction, not exhibiting any external marks of disease; neither did any material morbid appearances present immediately on opening the chest and abdomen. There were a few red patches upon the peritonæal coat of the stomach; the intestines externally shewed inflammation, though not to a high degree.

The stomach, previously tied at both ends, was taken out of the body; it contained about half a pint of fluid, of a reddish brown colour; the interior of the stomach, and also of the œsophagus, shewed high inflammation; the villous coat of the stomach was destroyed, and numerous portions of it, which had separated, were found in the fluid; other portions of different sizes, some about the size of a shilling, and isolated; others larger, still adhered. The parts attached were of an ash colour; felt hardish, as if corroded by a strong caustic. Upon *all* these portions of membrane, a white powder was both visible to the eye, and palpable to the touch.

The contents of the stomach were preserved, and submitted to chemical analysis.

The liver, the lungs, and the heart, appeared sound and free from inflammation; a few ounces of water were found in the pericardium. The internal surface of the intestines, so far as they were examined, was highly inflamed; the intestinal canal was not, however, examined through its whole course; many circumstances, unnecessary to mention, mention, concurring to prevent the dissection from being carried so far as could be wished.

The history of the case, the state of the œsophagus, stomach and bowels, and the appearance of the white powders, justified the opinion delivered before the coroner, that the deceased had died by poison, part of which was found in the stomach, and was most probably arsenic. Having a wish to know what the white powder actually was, I obtained, by agitating the portions of membrane in water, by washing and filtration, a portion of it in a dry state.

Three grains of this powder were boiled in six drachms of water, but did not dissolve; to this fluid was added a solution of fifteen grains of the sulphate of copper, in an equal quantity of water; the union produced a mixture of a sky blue colour. A similar compound made with arsenic in the shop, was of a fine green.

Three grains of the powder with two of charcoal and two drops of oil were mixed together, and after being secured betwixt two plates of brightened copper, were submitted to a red heat five minutes, but no white stain was perceivable. The fluid found in the stomach (with the washings, &c.) was boiled in a Florence flask, filtered, and suffered to cool. To a portion of this fluid a little lime water was added, and a slight white cloud only was perceived. A similar result took place on the addition to other portions of solutions of kali and sulphate of copper. The powder effervesced with sulphuric acid, and appeared to me to be white sand, with which, I have since been informed, it is common to adulterate arsenic sold in this country for farming purposes. To prove this, I made an experiment with two scruples, obtained from a druggist, and found it contained 25 grains of sand and chalky matter. The arsenic in this case was obtained by the young woman at a shop in Selby.

The preceding statement appears to me to be worthy of record in your useful publication, as a strong confirmation of the opinion delivered by Dr. Bostock, on the memorable trial at Lancaster, viz. that a person may be destroyed by taking poison; and yet, in consequence of vomiting, purging, and taking diluents, none can be detected after death. The unexpected result of this investigation, may also prevent others from forming a decisive opinion from appearances only, particularly upon occasions where the life of a fellow-creature will principally depend upon medical evidence. In the case published by Dr. Yelloly, in the 20th Number of the Edinburgh Medical Journal, the patient having taken arsenic, was attacked with vomiting, purging, &c. and drank a large quantity of warm water; after death, some white powder was found in the stomach, which was supposed to be arsenic. It was not however chemically examined. In the above related case, the quantity of sand found, could not have been less than two scruples, and the quantity of arsenic consequently considerable; yet, though she died in a few hours, none could be detected in the stomach. So far as an inference can be drawn from a solitary case, it appears from the above, that the common practice of giving diluents, if continued a long time, upon the supposition of the poison remaining in the stomach, must frequently do harm, (particularly in cases where a soluble substance, as blue and white vitriol, has been taken) by irritating the already too much irritated stomach unnecessarily; and that it would be more rational and consonant to the improved state of medical knowledge, after the stomach has been well emptied by an emetic, and a moderate use of diluents, to adopt such means as are likely to subdue the inflammation produced, and to administer such remedies as will produce quiescence in the stomach and bowels. It is much to be regretted, that there are so few histories of cases of death by, or recovery from, the effects of poison, in your valuable publication. On looking over it from its commencement, I cannot find more than two or three at most, where mineral poison had been taken. It appears to me, that details of such cases would be useful to many of your readers, who from locality of situation, cannot always confer with their medical brethren in cases of emergency, and who, from the high price of books, cannot always obtain what information is already published. Your widely circulated Journal furnishes the proper medium for the communication of any new facts in the posession of, or observations made by any of your numerous Correspondents, who, I hope, as it is a subject not yet sufficiently investigated, will favour your Readers with the results of their experience.

I am, &c.

THOMAS JONES,

Member of the Royal College of Surgeons, London.

*Howden,*

*April* 2, 1810.

